# Regulatory mechanisms of microRNAs in lung cancer stem cells

**DOI:** 10.1186/s40064-016-3425-5

**Published:** 2016-10-10

**Authors:** Tao Fan, Wei Wang, Boyou Zhang, Yao Xu, Lei Chen, Shize Pan, Hao Hu, Qing Geng

**Affiliations:** Department of Thoracic Surgery, Renmin Hospital of Wuhan University, Wuhan, 430060 China

**Keywords:** MicroRNAs (miRNAs), Lung cancer stem cells (LCSCs)

## Abstract

Increasing evidence suggests that cancer stem cells (CSCs) are a key occurrence in the process of many human cancers. Lung cancer is the most common aggressive malignancy and cause of cancer death worldwide. The research on lung cancer stem cells has been highlighted for many years. Lung CSCs seem to play a major role in lung cancer metastasis, drug resistance and tumour-self-renewal. MicroRNAs (miRNAs), a class of newly emerging small noncoding RNAs that act as post-transcriptional regulators of gene expression, have been demonstrated to serve as a vital player in fine-tuning a number of biological activities ranging from embryogenesis to programmed cell death as well as tumourigenesis. In recent years, several miRNAs have been highlighted to be specifically expressed in CSCs. The miRNA profile of CSCs is remarkably different from non-stem cancer cells. As such, many miRNAs have been shown to regulate self-renewal and differentiation properties of CSCs. In this review, we present the latest findings on miRNAs that regulate the tumour microenvironment of lung CSCs with the goal to prompt the development of novel therapeutic strategies for patients with lung cancer.

## Background

Lung cancer is the leading cause of cancer deaths and the most common aggressive carcinoma worldwide (Ferlay et al. 2010; Papagiannis 2004). The research on oncotherapy has been highlighted for more than half a century. Accurately understanding the molecular biology of cancer is the only possibility for conquering lung cancer. During the past 20 years, the discovery and study of miRNAs has provided a new approach for the treatment of cancer. Since Lee et al. (1993) and Reinhart et al. (2000) first identified miRNAslin-4 and let-7controlling the developmental timing in nematode *Caenorhabditiselegans*, extensive studies have been conducted to discover the functional role of miRNAs in multiple biological activities, ranging from embryonic development to cell death (Fabian and Sonenberg 2012; Pauli et al. 2011; Rinn and Chang 2012), and from cancer formation to its development (Li et al. 2009; Wang et al. 2013b; Ohashi et al. 2011; Pang et al. 2010c; Rachagani et al. 2010; Kurisetty et al. 2014; Serpico et al. 2014; D’Ippolito and Iorio 2013).

Since Mackillop (Mackillop et al. 1983) first put forward the hypothesis of cancer stem cell theory, the overwhelming mass of research has infiltrated into the characterization of cancer stem cells (CSCs) from different tumours (Chhabra and Saini 2014). The cancer stem cell hypothesis is based on a theory that cancer has a hierarchical cell structure in which only a subpopulation of cells, termed cancer stem cells (CSCs), which have the ability to regulate self-renewal and differentiation of cancer cells, are able to initiate tumour formation. In addition, they also fine-tune tumour metastasis by altering genetic signalling pathways (Pang et al. 2010a; Hermann et al. 2007). Lung CSCs, which have the characteristics of self-renewal and differentiation, were first isolated from the junction between the rat bronchioles and alveolar ducts by Kim in 2005.

miRNAs are short (20- to 24-nucleotide), small and non-coding single-stranded RNA molecules that can inhibit gene expression by conducting target mRNA degradation or translational repression. Its molecular mechanism is that primary miRNAs (primiRNAs), which are transcribed by RNA polymerase II or III with 5′-end caps and 3′-end poly-A tails (Borchert et al. 2006; Lee et al. 2004; Cai et al. 2004), bind to the 3′-untraslated regions (3′UTRs) or the open reading frames of target genes to influence expression of mRNA. miRNAs regulate gene expression. It has been demonstrated that miRNAs inhibit gene translation in a proliferating state but stimulate it in a quiescent state (Vasudevan et al. 2007; Bartel 2004; Garzon et al. 2009). It can be summarized that miRNAs participate in a series of biological processes including cell proliferation, apoptosis, the immune response and differentiation of CSCs (Dumortier et al. 2013; Hwang and Mendell 2006; Chivukula and Mendell 2008; Esau et al. 2004; Zhao et al. 2010; DeSano and Xu 2009; Perera and Ray 2007) by intervening with biological signals of transcription, nuclear processing, exportation, and cytoplasmic processing, as well as inhibiting or activation translation (Bartel 2004; Garzon et al. 2007, 2009; Liu and Tang 2011). Abundant expression of miRNAs has also been found in CSCs. Since Lee et al. (1993), Reinhart et al. (2000) first identified miRNAs regulating stem cells in *Caenorhabditiselegans*, cancer research has stepped into a new stage in recent years. If the CSC hypothesis is true, then deletion of CSCs would be a perfect treatment, and the aberrant expression profile of miRNAs may be the cause of the initiation and development of the tumour (Lobo et al. 2007). Therefore, regulating expression of miRNAs would be a new target to treat cancer. This aspect, however, needs further study.

### miRNAs IN LUNG CSCs

**Fig. 1:**
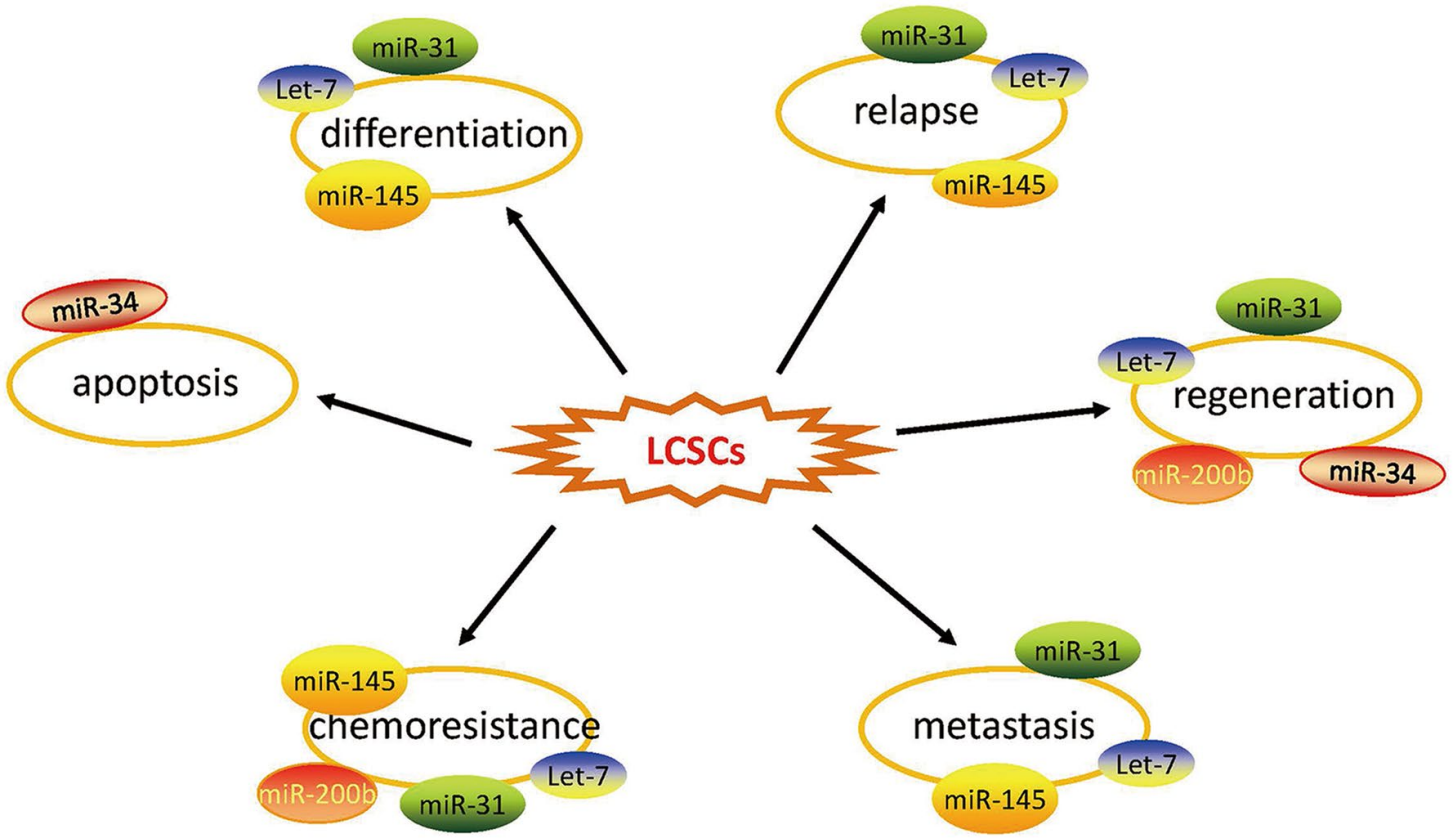
The different miRNAs regulate properties of lung cancer stem cells (LCSCs). By targeting a variety of downstream signalling pathways, several miRNAsact synergistically to regulate several key biological properties of LCSCs including differentiation, relapse, regeneration, metastasis, chemoresistance and apoptosis

An increasing number of miRNAs have been confirmed to be involved in self-renewal and metabolism of lung CSCs. miRNA-145 was found to be expressed at low levels in lung adenocarcinoma (LAD)-associated CSCs and was further validated to inhibit the proliferation of LAD-CSCs and be negatively correlated with the levels of Oct4/Sox2/Fascin1 in LAD patient specimens (Chiou et al. 2012; Zhang et al. 2011; Hu et al. 2014). In two other studies on lung adenocarcinoma CSC, let-7and miR-31 were significantly down-regulated in side population (SP) cells, which are an enriched source of CSCs, compared to non-SP cells (Hua et al. 2012). Interestingly, these two miRNAs play opposing roles to maintain a balance between differentiation and quiescence of LAD-CSCs. A previous study showed that miRNA-200b is significantly down-regulated in CD133^+^/CD326^+^cells, which exhibit characteristics of CSCs derived from docetaxel-resistant LAD cells. These findings strongly suggest that aberrantly expressed miRNAs, such as miR-29ab, miR-183, and miR-127-3P, play significant roles in regulating the CD133+/CD326+ subpopulation of cells (Lin et al. 2012). Therefore, miRNAs could substantially affect the biobehaviours of LCSCs, including cell cycle, proliferation, apoptosis, immune response and differentiation, by controlling the signalling pathways of LCSCs. Here, we present the latest findings on miRNAs that regulate the tumour microenvironment of lung CSCs to prompt the development of novel therapeutic strategies for patients with lung cancer (Table 1). Figure 1 shows the potential therapeutics of miRNAs in LCSCs.

**Table 1:** Regulatory miRNAs involved in LCSCs

MicroRNAs	Source	Tumor type	Correlated factors	Target genes	Cell surface markers	Signaling pathway	References
miR-145	Human lung cancer tissues	Adenocarcinoma	Sox2, fascin	Oct4	SP/CD133	Oct4/sox2/fascin	Chiou et al. (2012), Zhang et al. (2011), Hu et al. (2014),
miR-31	A549cell lines/side population cells	Adenocarcinoma	G0/G1 phase	G0/G1 phase	SP/CD133/326	Unknown	Hua et al. (2012), Lin et al. (2012)
miR-7	A549cell lines/side population cells	Adenocarcinoma	G1/S phase	G0/G1 phase	SP/CD133/326	Unknown	Hua et al. (2012), Lin et al. (2012)
miR-34a	A549cell lines/H460/H1299/mice	Non-small cell lung cancer	p53/BCL2	p53/BCL2	CD44/CD133	p53/Notch1/Nontch2	Shi et al. (2014), Bommer et al. (2007), Li et al. (2009), Pang et al. (2010b), Balca-Silva et al. (2012)
miR-200b	SPC-A1/H1299/human lung cancer tissues	Adenocarcinoma	HDAC1/Oct-4/SOX-2/Bmi-1	Suppressor of zeste-12 (Suz-12)	CD133/326	The HDAC1/miR-200b/Suz-12-E-cadherin signaling	Chen et al. (2014)

### miRNA-145 regulates lung CSCs

**Fig. 2:**
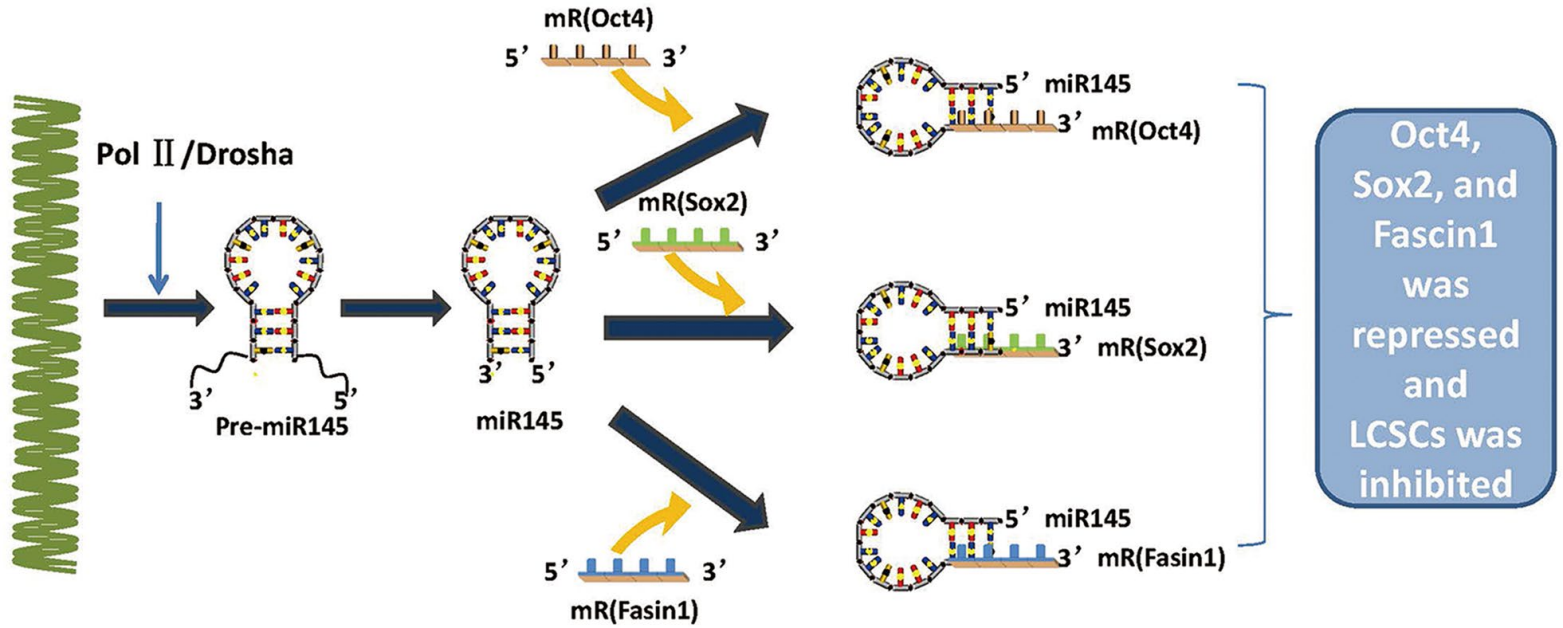
miR-145 is transcribed by RNA polymerase II (Pol II) into long primary miRNA145 transcripts, which are cleaved in the nucleus by the RNase III enzyme Drosha, resulting in a hairpin precursor form called pre-miRNA. Pre-miRNA is exported from the nucleus to the cytoplasmby exportin 5 and is further processed by the enzyme Dicer, which produces a transient miRNA duplex that includes a mature miR-145. miR-145 targets sites of the 3′UTRs of Oct4, Sox2, and Fascin1 and represses Oct4, Sox2, and Fascin1 to inhibit the properties of LCSCs

miRNA-145 is an important regulator of tumourigenesis. Previous research by Cho WC demonstrated that miRNA-145 inhibited cell proliferation of human lung adenocarcinoma by targeting EGFR and NUDT1 (Cho et al. 2011). Lu et al. (2014) further found that mirRNA-145 functions as a tumour suppressor and targets two oncogenes, ANGPT2 and NEDD9, in renal cell carcinoma. In recent years, it has been shown that mirRNA-145 is down-regulated in lung adenocarcinoma tissues and negatively correlates with the expression of Oct4 (Yin et al. 2011), which is expressed in lung CSCs (CD133^+^cells) (Hu et al. 2014). The precise molecular mechanism of mirRNA-145 in lung CSCs is not known. A previous study indicated that miRNA-145 reduced the capacity of proliferation, invasion and tumour sphere growth in lung adenocarcinoma-initiating cells. Epithelial-mesenchymal transition (EMT), which regulates a dedifferentiation programme that converts adherent epithelial cells to individual migratory cells, plays an important role in regulating embryonic development, cancer migration, and tumour metastasis (Kalluri and Weinberg 2009; Thiery 2002), which is similar to CSCs (Pirozzi et al. 2011). Dai et al. (2013) confirmed that the EMT process of colorectal cancer cells was regulated by Oct4, an important transcriptional factor that has been proposed as a biomarker of cancer-initiating cells (CICs). Hu et al. (2014) further verified the amazing effect of miR-145 on properties of lung CSCs and EMT in vivo by regulating Oct4. It has been reported that cationic polyurethane-short branch polyethylenimine (PU-PEI) exhibits high transfection efficiency with relatively low cytotoxicity (Hung et al. 2009; Liu et al. 2009) and has been shown to be a deliverer for overexpression of Oct4 and SirT1 in the process of reprogramming aged retinal pigmented epithelium cells into progenitor-like cells and rescuing light-induced retinal cell loss and dysfunction (Peng et al. 2011). Guang-Yuh and his colleagues found that LAD patients with a microRNA-145^low^, Oct4^high^, Sox2^high^, or Fascin1^high^ phenotype had a poor prognosis and outcome, and microRNA-145 was proven to be the most relevant marker (Chiou et al. 2012). As a vehicle formicroRNA-145 delivery, PU-PEI mediates miR-145 overexpression and inhibits the properties of LAD-CSCs by repressing its downstream targets, Oct4, Sox2, and Fascin1 (Chiou et al. 2012), which provides a novel miRNA-based approach for the treatment of LAD by artificially enhanced expression of microRNA-145 (Fig. 2).

### MicroRNA-31 and let-7 regulate lung CSCs

**Fig. 3:**
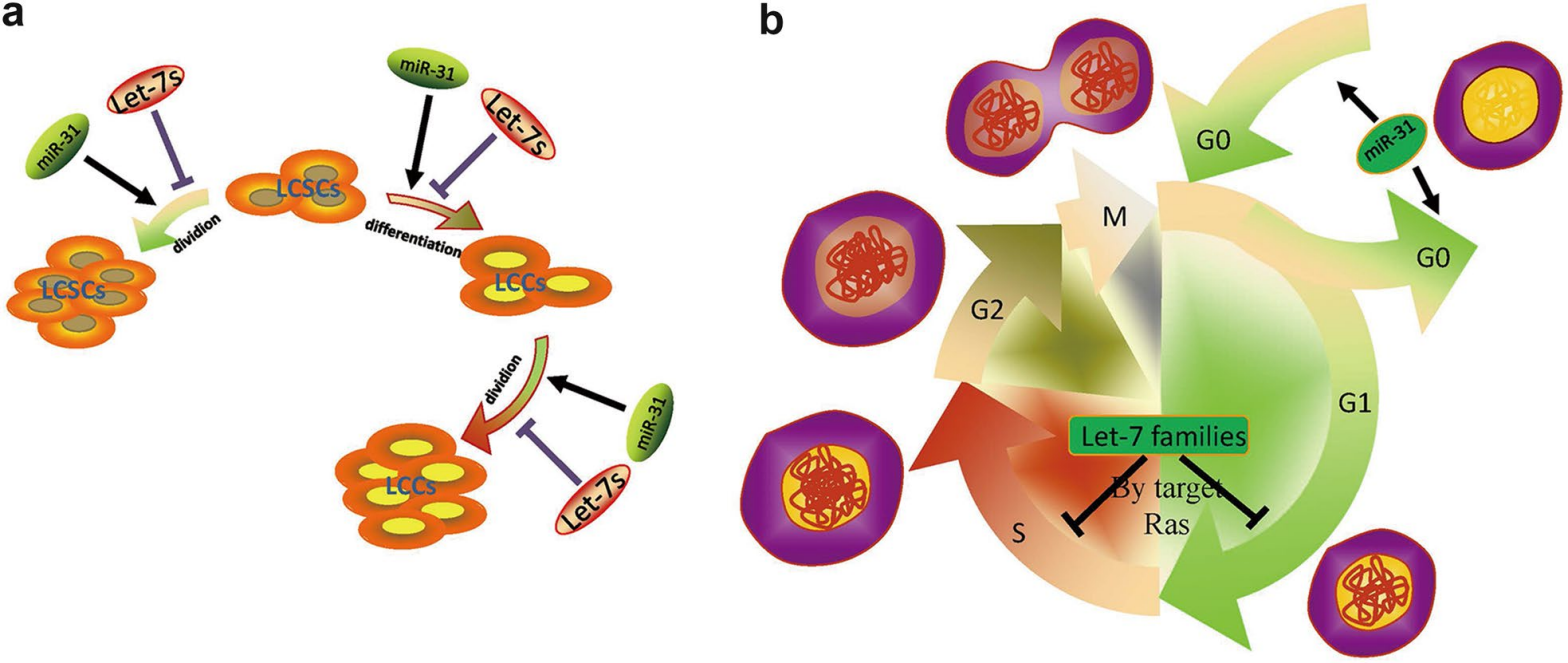
As is shown in **a**, let-7 and miR-31 are regulators for maintaining the balance between differentiation and quiescence for LCSCs. Cell cycle studies further revealed that increasing miR-31 could enhance the proliferation of LCSCs by speeding up the cell cycle in the G0/G1 phase, whereas reduced let-7 induced cell proliferation of LCSCs by accelerating the G1/Sphase transition (**b**)

Encoded by a single genomic locus, microRNA-31 is expressed in a variety of tissues and cell types (Grimson et al. 2007; Landgraf et al. 2007). Altered expression of miR-31 has been verified in various human tumours (Valastyan and Weinberg 2010). It is reported that miR-31 levels are inversely associated with the propensity to suffer metastatic relapse in primary human breast tumours (Valastyan et al. 2009b). Similarly, down-regulation of miR-31 was observed in human bladder carcinomas with an invasive phenotype (Wszolek et al. 2011). Valastyan et al. (2009a) has stated that miR-31 impairs more than three distinct steps of the invasion–metastasis cascade: local invasion, one or more early post-intravasation events (intraluminal viability, extravasation, and/or initial survival in the parenchyma of distant tissues), and metastatic colonization (the outgrowth of micrometastases into macroscopic secondary lesions). The let-7 family, one of the most highly up-regulated families of miRNAs, consists of 12 members located in genomic locations frequently deleted in human cancers (Calin et al. 2004). The let-7 family targets several key genes in the PI3K/AKT/insulin pathway during cardiac maturation (Kuppusamy et al. 2015). The early research on let-7 in the field is about energy metabolism (Zhu et al. 2011; Frost and Olson 2011). In recent years, it has been reported that let-7 was down-regulated in several cancers (Johnson et al. 2005; Mayr et al. 2007; Sampson et al. 2007). Johnson et al. (2005) found that expression of let-7 RNA is reduced in non-small cell lung cancer patients and is associated with poor prognosis. In addition, overexpression of let-7 reduces tumour burden in a K-Ras murine lung cancer model (Sampson et al. 2007). SP cells are a rare cell population that are an enriched source of stem cells in some normal tissue and intumours, such as glioma, colorectal, ovarian cancer and lung cancer tumours (Kondo et al. 2004; Brown et al. 2007; Szotek et al. 2006; Ho et al. 2007; Larderet et al. 2006). Under differentiating conditions, SP cells can differentiate into non-SP cells and lose their stem-like properties (the ratio of SP gradually decreases with culture time) (Hua et al. 2012). It has been confirmed that microRNA-31 and let-7 are significantly down-regulated in lung SP cells (lung CSCs). Interestingly, Hua et al. (2012) found that low expression of let-7 was the key to the preservation of their ability to proliferate, and low expression of microRNA-31 was necessary for their undifferentiated status to persist. It can be confirmed that the reduced expression of let-7 and microRNA-31were critical for SP cells to preserve their stemness. MicroRNA-31 acts as an oncogenic gene, while let-7 functions as a lung cancer suppressor microRNA (Johnson et al. 2007; Liu et al. 2010), which suggests that miR-31 and let-7 have opposite functions on lung CSCs. Antisense oligonucleotide transfection experiments have supported this view by cell cycle research that showed that reduced miR-31 could inhibit cell proliferation by a cell cycle arrest in the G0/G1 phase, whereas reduced let-7 induced cell proliferation by accelerating the G1/S phase transition (Hua et al. 2012; Fig. 3). As regulators for maintaining the balance between differentiation and quiescence for SP cells, let-7 and microRNA-31 would be novel endogenous lung CSC targets to treat lung cancer.

### miRNA-34a regulates lung CSCs

**Fig. 4:**
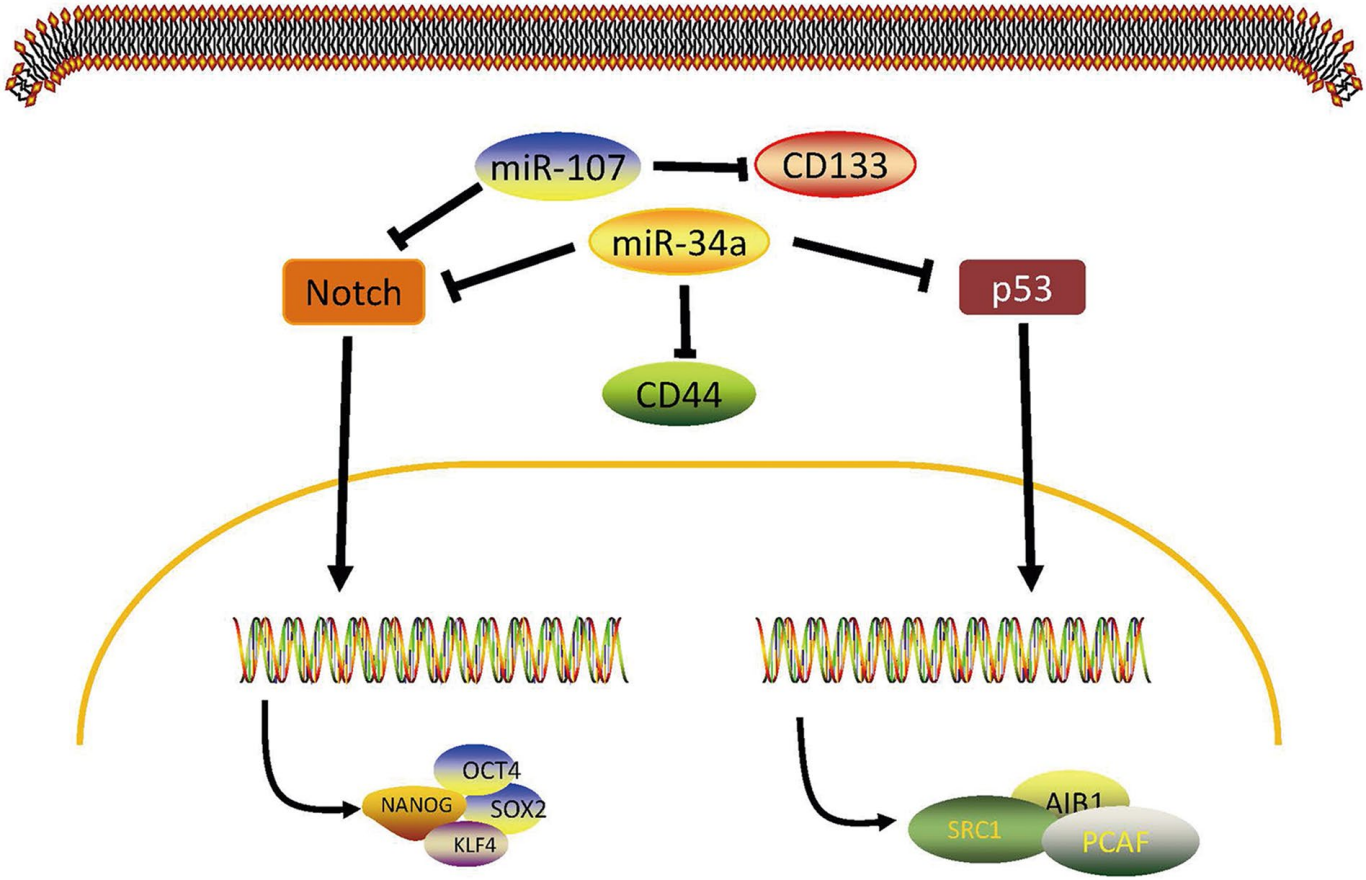
miR-34a is a direct transcriptional target of p53 and is a negative regulator of the tumourigenic properties of CD44^hi^ lung CSCs. As is shown above, over expression of miR-34a could inhibit regeneration of LCSCs by repressing CD44 and the Notch and p53 pathways

The miRNA-34 family, which is composed of miR-34a, miR-34b, and miR-34c, is confirmed to be involved in the p53 and Notch signalling pathways (Shi et al. 2014; Bommer et al. 2007; Li et al. 2009; Pang et al. 2010b; Balca-Silva et al. 2012; Fig. 4). Pang et al. have demonstrated that miRNA-34a binds to the 3′-untranslated regions of Notch1 and Jagged1. miRNA-34a is a tumour suppressor gene (Chen and Hu 2012) and inhibits abnormal cell growth, such as that in pancreas, melanoma, lung, breast, prostate, osteosarcoma, and gliomatumours (He et al. 2009; Poellinger and Lendahl 2008; Yang et al. 2013; Liu and Tang 2011; Yan et al. 2012; Wiggins et al. 2010; Bandi and Vassella 2011). However, miR-34b/c, which has been identified in cases of malignant melanoma and glioma, may have the opposite function (Wu et al. 2013).With the research on the CSC hypothesis, the miR-34 family has now been shown to affect major properties of CSCs. Liu et al. (2011) have shown that miRNA-34a can inhibit prostate CSCs by directly repressing CD44. In another instance, enforced expression ofmiR-34 inhibited the self-renewal capacity of CD44C/CD133C pancreatic CSCs through the directdown-regulationofBcl-2and Notch signalling pathways (Wang et al. 2006). Furthermore, miRNA-34a was demonstrated to inhibit NSCLC cell holoclone formation and clonogenic expansion in vitro and even to repress tumour regeneration in vivo (Shi et al. 2014), which are due to its effects on stem-like NSCLC (NSCLC-CSCs). Further study verified that enhanced expression of miRNA-34a in CD44^hi^ H460 cells greatly inhibited their tumour-regenerating activity. On the contrary, antagonists of miR-34a dramatically promoted tumour regeneration in CD44^lo^H460 cells. Similarly, these observations are confirmed in prostate and lung cancer (Wang et al. 2013a). In addition, the lower expression of miR-34a was considered to be a key aetiologic factor contributing to the aggressive behaviour of lung cancer stem cells (CSC), and thus, those features were mitigated by exogenous delivery and restoration of miR-34a activity (Basak et al. 2013). Taken together, as a regulator of the tumour-initiating capacity in NSCLC-CSCs, restoration of miR-34 levels may be used as a cancer therapeutic by down-regulating the Notch family members or target genes. The replacement of oncosuppressor miRNAs provides an effective strategy against tumour heterogeneity, and the selective RNA-based delivery system seems to be an excellent platform for a safe and effective targeting of tumours (Misso et al. 2014). Future work will aim to further clarify the underlying mechanisms and intervene in its signalling pathways to find a cure for cancer.

### miRNA-200b regulates lung CSCs

**Fig. 5:**
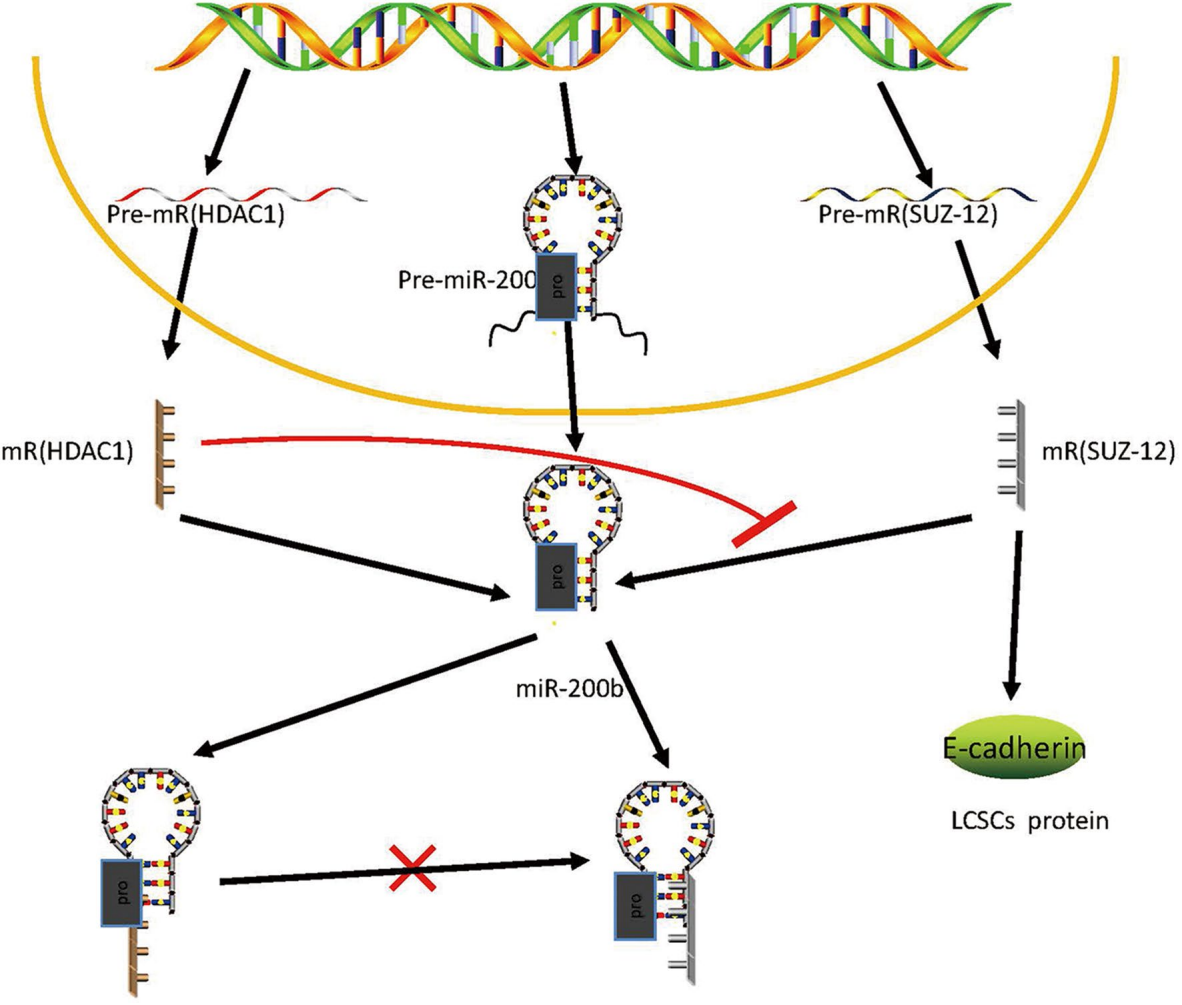
Suppressor of zeste-12 (Suz-12) was identified as a direct and functional target of miR-200b. Histone deacetylase (HDAC) 1 repressed miR-200b through a specificity protein (Sp) 1-dependent mechanism in which HDAC1 and Sp1 could bind to the miR-200b promoters (pro). Restoration ofmiR-200b by HDAC1 repression significantly suppressed CSC formation and reversed chemoresistance of CSCs by reducing Suz-12-E-cadherin

Located at the miRNA-200b/c/429 gene cluster, miRNA-200b, as an important member of miRNA-200 families, has the capability of repressing CSC growth and reversing the EMT phenotype of CSCs (Iliopoulos et al. 2010; Sun et al. 2012). The miRNA-200 family plays a key role in determining the epithelial phenotype by targeting zinc-finger transcriptional repressors ZEB1 or ZEB2 (Comijn et al. 2001; Gregory et al. 2008; Park et al. 2008). In recent years, miR-200b was demonstrated to be a key regulator of chemoresistance and restoration and has been shown to significantly reverse chemoresistance of docetaxel (DTX)-resistant (lung adenocarcinoma) LAD cells by inducing cell cycle arrest and apoptosis enhancement (Feng et al. 2012). Chen et al. (2014) first showed that miR-200b functions as a tumour suppressor in LAD CSCs both in vitro and in vivo. With further study, they demonstrated that histone deacetylase 1 (HDAC1) is involved in silencing of miR-200bthrough a Sp1-dependent mechanism. Inhibition of HDAC1 could induce miR-200b expression, which significantly suppresses maintenance of CSCs and reverses chemoresistance of CSCs by regulating Suz-12-E-cadherin signalling (Chen et al. 2014). CSCs have already been associated with chemotherapeutic failure in a variety of solid tumours. HDAC1/miR-200b/Suz-12/E-cadherin, which is involved in the regulation of CSC self-renewal, maintenance, tumourigenicity, growth and chemoresistance, may be the key regulatory network in regulating CSC maintenance and chemoresistance in human LAD cells (Fig. 5). This discovery will provide a novel strategy for reversing chemoresistance of human LAD.

## Conclusions and perspectives

By targeting mRNAs for translational repression or degradation, miRNAs play important regulatory roles in animals and plants. As one of the most abundant classes of gene regulatory molecules in organisms, miRNAs likely influence the output of many protein-coding genes (Bartel 2004). There is mounting evidence to suggest that miRNAs are involved in many fundamental cellular processes such as cell differentiation, proliferation, apoptosis, carcinogenesis and cancer progression (Winter et al. 2009; Joshi et al. 2014). Correlated with characteristic clinicopathological parameters in cancer subtypes and existing not only in tissue but also in body fluids, microRNAs are potential biomarkers for different cancer subtypes classified by origin, histology, or chemosensitivity (Lu et al. 2005; Gilad et al. 2008; Kishikawa et al. 2015; Hollis et al. 2015). Due to their characteristic nature, microRNAs have a potential to be used for the development of diagnostics, prognostics, and targeted therapeutics.

In this review, the putting forward of the CSC theory gives researchers new ideas for recognizing the initiation of cancer and the regulation of cellular signalling in cancer and CSCs. Doubtless, CSCs do exist in tumours and drive tumour growth. Deeper research into CSCs will be a key to overcoming cancer. We have presented several regulatory mechanisms in CSCs, which mainly include miRNAs. In fact, since miRNAs were discovered in the 1990s, they have been increasingly confirmed to play a critical role in carcinogenesis and cancer regulation. As there have been aberrant miRNA expression profiles identified in lung cancer, miRNAs could potentially be used as biomarkers in the diagnosis and classification of lung cancer instead of helical computed tomography (CT) or even pathological examination. Despite the fact that inspiring progress has been achieved in miRNA-mediated gene activation, many questions remain to be further elucidated. For example, we do not know if different miRNAs interfere with each other in regulating tumour genesis and cancer progression. Moreover, the research in the CSC field is at an early stage. The specificity and accuracy of lung CSC-associated miRNA signatures need to be studied further.

This review aimed to summarize recent achievements in research on miRNAs regulating lung cancer stem cells. Accumulating evidence indicates that miRNAs play critical roles in lung CSCs. They can effectively control lung cancer stem cell cycles, proliferation, apoptosis, immune response and differentiation by regulating the signalling pathways of LCSCs. miR-145 can suppress the cancer stem cell-like properties of LCICs and the EMT process by targeting Oct4. Additionally, let-7 and microRNA-31 are critical for SP cells to preserve their stemness, as it was revealed that reduced miR-31 could inhibit cell proliferation by a cell cycle arrest in the G0/G1 phase, whereas reduced let-7 induced cell proliferation by accelerating the G1/S phase transition.miR-34a over expression inhibits NSCLC cell holoclone formation and clonogenic expansion in vitro and, importantly, tumour regeneration in vivo. The novel HDAC1/miR-200b/Suz-12/E-cadherin pathway was confirmed to play an essential role in regulating maintenance, tumourigenicity and chemoresistance of CSCs in human LAD. However, further studies are needed for a more detailed understanding of the role of miRNAs in the biology of lung CSCs. The future further insights into the molecular mechanisms of tumourigenesis will provide novel treatment strategies for patients with lung CSCs.
